# Supplementary material for: Organic Nanoparticles Based on D-A-D Small Molecule: Self-Assembly, Photophysical Properties, and Synergistic Photodynamic/Photothermal Effects
Source: Materials (Basel). 2022 Jan 10;15(2):502. doi: 10.3390/ma15020502 (PMC8781609; doi:10.3390/ma15020502)
Supplement: Supplementary file 1 [file materials-15-00502-s001.zip › materials-1487095-supplementary.pdf]

materials

Supplementary Materials

Organic Nanoparticles Based on D-A-D Small Molecule: Self-Assembly, Photophysical Properties, and Synergistic Photodynamic/Photothermal Effects

Experimental Sections

Materials and Methods

3,4,9,10-perylenetetracarboxylic dianhydride (PDI) (Meryer Chemical, 98%), 2-(2-(2-ethoxyexthoxy) ethoxy) ethanol (Aladdin, 98%) and Triphenylamine (TPA) (Geo Chemical, 98%) were directly used without further purification. Tetrahydrofuran (THF), ethanol and N, N-dimethylformamide (DMF), Tetrahydrofuran (THF) were dried by vacuum distillation before use. A549 cells were obtained from Cell Bank, Chinese Academy of Sciences. DCFH-DA was purchased from Maclin Biochemical Co., Ltd. DAPI was obtained from Beyotime Biotechnology (China), and Calcein-AM and PI were purchased from Shanghai Aladdin (China). Other reagents involved in the experiment were purchased from Shanghai Aladdin (China) and used directly in the reaction without further purification.

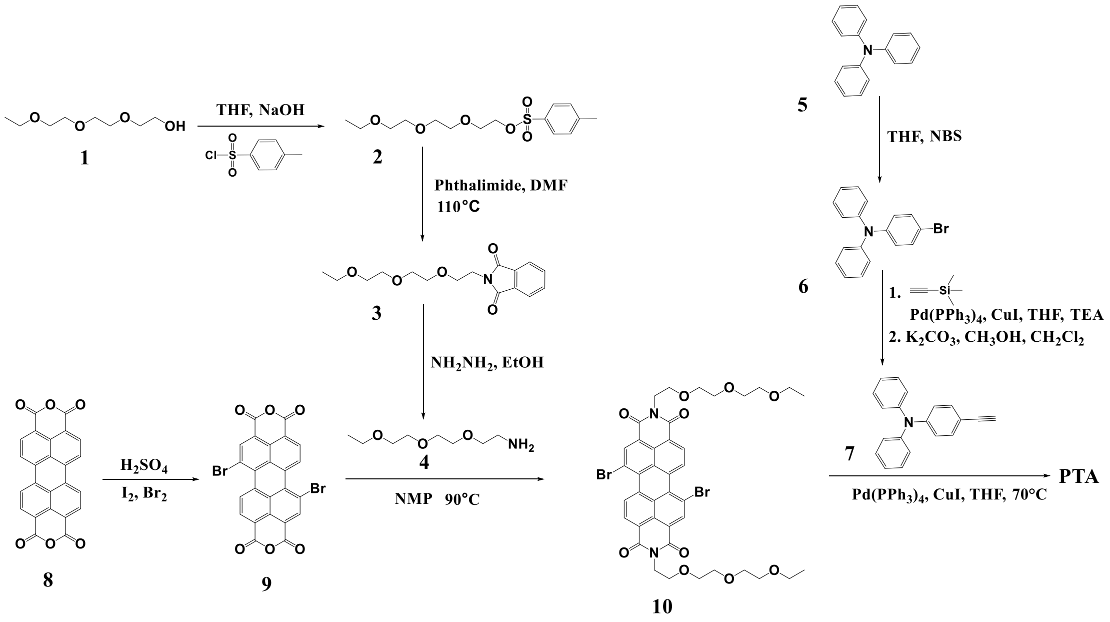

Scheme S1. The synthetic routes of the intermediates 4 and 9 and precursors 7 and 10.

Synthesis of 2-(2-(2-ethoxyethoxy)ethoxy)ethylamine (4) [1]

2-(2-(2-ethoxyexthoxy) ethoxy) ethanol (1) (5.00 g, 28.00 mmol) was dissolved in THF (15.00 mL) placed in an ice bath. Then a solution of NaOH (2.00 g, 50.00 mmol) in water (18.4 mL) was added to the mixture under nitrogen. A solution of THF (15 mL) containing *p*-toluene sulfonyl chloride (8.00 g, 42.00 mmol) was added dropwise within 20 min. After heated to 35 °C for 2 h, the reaction mixture was diluted with ethyl acetate (100 mL) and then washed with 1 M NaOH (3 × 50 mL) and water (2 × 30 mL), respectively. The solvent

was dried with anhydrous Na_2SO_4 and removed under reduced pressure to afford yellow liquid (2) (15.61 g, 84%), which was used without further purification.

Under nitrogen atmosphere, 2-(2-(2-ethoxyethoxy) ethoxy) ethyl tosylate (2) (5.00 g, 15.06 mmol) and potassium phthalimide (3.15 g, 17.00 mmol) were dissolved in DMF (10 mL). After stirring at 110 °C for 3 h, the solvent was concentrated under reduced pressure. The residue was diluted with ethyl acetate and washed with 1M NaOH and water, respectively. The organic solution was dried with anhydrous Na_2SO_4 and removed under reduced pressure. The crude product (3) was then dissolved in 6.4 mL of a hydrazine monohydrate/ethanol (1/1, v/v) mixture. After heated to 110 °C under N_2 protection for 12 h, the mixture was cooled to room temperature and extracted with diethyl ether. The combined organic layer was concentrated under reduced pressure to afford compound (4) as a yellow oil (1.60 g, 60%), which was used in the next reaction without further purification.

Synthesis of 4-bromo-N,N-diphenyl aniline (6) [2]

A two-neck flash equipped with triphenylamine (1.00 g, 4.10 mmol) in $CHCl_3$ (20 mL) under N_2 protection at 0 °C was charged with N-bromosuccinimide (0.80 g, 4.51 mmol). After stirred at room temperature for 12 h, the mixture was washed with water, dried over anhydrous $MgSO_4$ and concentrated via rotary evaporator. The target product (6) (1.06 g, 79 %) was obtained by recrystallization in MeOH. ^{1}H NMR (400 MHz, $CDCl_3$) δ 7.33 (d, *J* = 8.6 Hz, 2H), 7.26 (dd, *J* = 7.6, 4.3 Hz, 4H), 7.08 (d, *J* = 7.6 Hz, 4H), 7.04 (s, 2H), 6.95 (d, *J* = 8.7 Hz, 2H) ppm.

Synthesis of 4-ethynyl-N,N-diphenylaniline (7) [3]

Trimethylsilyl acetylene (1.3 mL, 12 mmol) was added into anhydrous THF (10 mL) and TEA (10 mL) charged with compound 6 (0.40 g, 1.24 mmol), CuI (11.4 mg, 0.06 mmol) and $PdCl_2(dppf)_2$ (42.1 mg, 0.06 mmol) and the reaction was kept at 70 °C under N_2 for 3 h. After removing solvents, the residue was purified through column (silica gel) chromatography to afford the product as a light-yellow oil (0.271 g, 64 %). ^{1}H NMR (400 MHz, DMSO-d_6) δ 7.35–7.19 (m, 6H), 6.99 (dd, *J* = 13.2, 7.7 Hz, 6H), 6.82–6.71 (m, 2H), 0.14 (dd, *J* = 7.9, 6.5 Hz, 8H) ppm.

Next, the mixture of the compound 7 (0.395 g, 1.16 mmol) and K_2CO_3 (2.013 g) in MeOH (15 mL) and CH_2Cl_2 (15 mL) under N_2 atmosphere was stirred at room temperature for 3 h. The residue after solvent evaporation was purified through silica gel column chromatography with hexane/dichloromethane (4/1) as eluent to afford the desired product as a brown solid (0.290 mg, 93%), which was used without further characterization.

Synthesis of N,N-bis[2-(2-(2-Ethoxyethoxy)ethoxy)ethylane]-1,7-dibromoperylene - 3,4,9,10-tetracarboxylic acid bisimide (10) [4]

A two-neck flask equipped with compound (9) (1.00 g, 1.8 mmol) and compound 4 (0.96 g, 5.4 mmol) in N-methyl-2-pyrrolidone (NMP) (6 mL) was charged with propionic acid (1 mL) at 130 °C under N_2 atmosphere for 6 h. Then the reaction solution was poured into 50 mL water. The red precipitate collected by filtration was further purified by column (silica gel) chromatography (MeOH: DCM = 1:30) to afford a red solid 10 (1.27 g, 81%). ^{1}H NMR (400 MHz, $CDCl_3$) δ 9.25 (d, *J* = 8.1 Hz, 1H), 8.72 (s, 1H), 8.50 (d, *J* = 8.2 Hz, 1H), 4.43 (s, 2H), 3.90–3.39 (m, 12H), 1.14 (t, *J* = 6.9 Hz, 3H) ppm. ESI-MS: m/z Calcd for $C_{40}H_{40}Br_2N_2O_{10}$ $[M+H]^+$ 869.57, found 869.76.

Synthesis of 1,7-dibromoperylene-3,4,9,10-tetracarboxydianhydride (9) [5,6]

Perylene-3,4,9,10-tetracarboxylic dianhydride (8) (10.0 g, 25.50 mmol) was added to a three-necked round-bottom flask with concentrated sulfuric acid (80 mL). The resulting mixture was stirred at room temperature for 17 h. Subsequently, the reaction was heated to 85 °C and iodine (250.60 mg, 1.00 mmol) was added. After stirring for 1 h, bromine (2.50 mL, 33.15 mmol) was slowly added to the reaction mixture in a period of 2 h. After 20 h,

the reaction was cooled to room temperature, followed by the addition of 200 mL of ice water. The resulting red solid was collected by filtration, and then washed with sodium sulfite solution (5%, 300 mL) and water (250 mL) until the pH value was 7. The obtained compound was dried under reduced pressure to afford a red powder (12.6 g, 90.2%), which was used without further purification.

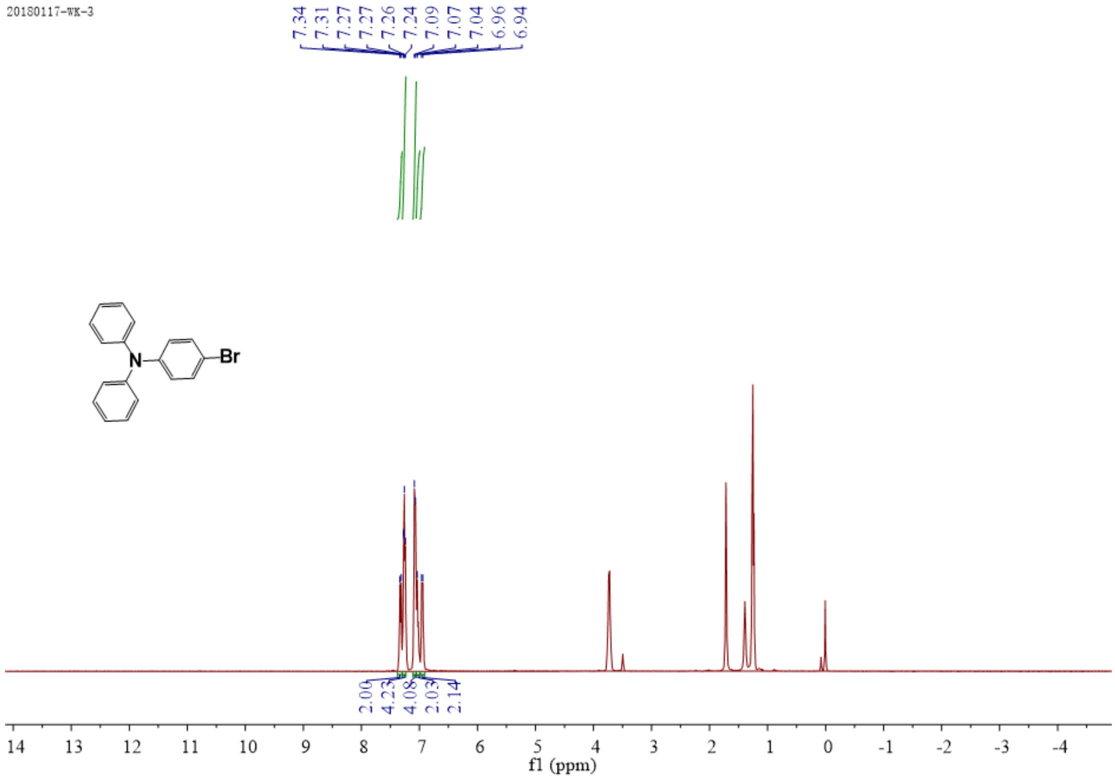

Figure S1. The ^{1}HNMR of structure 6

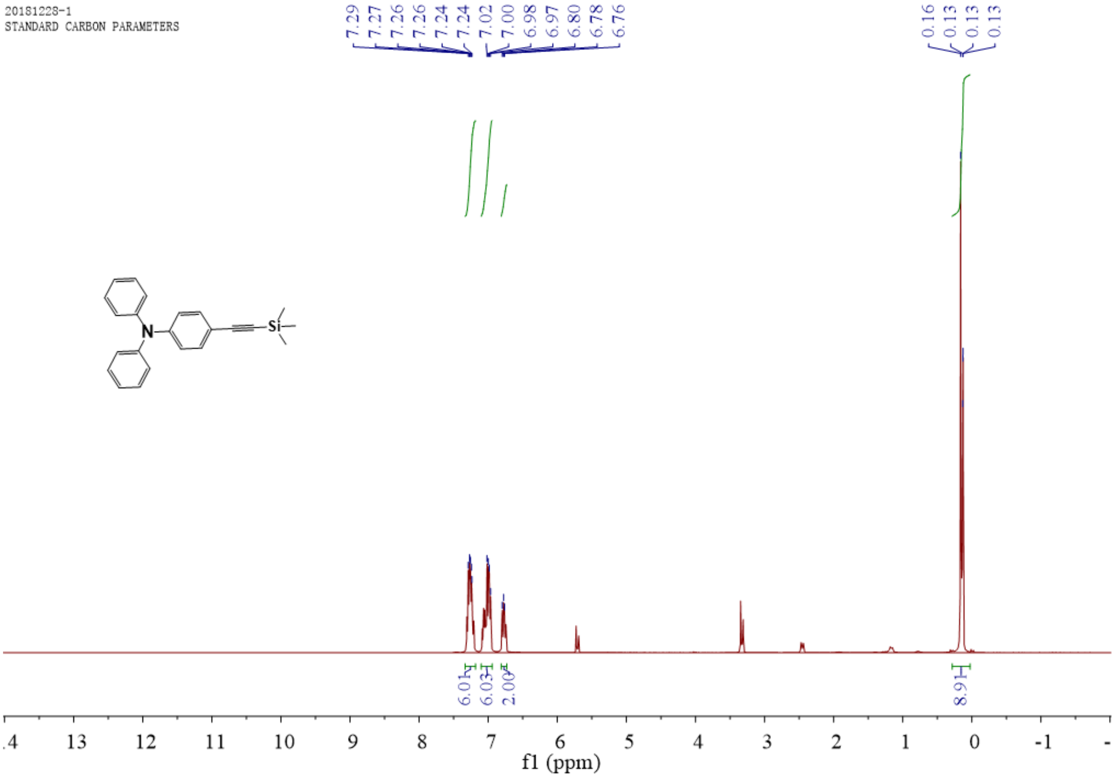

Figure S2. The ¹HNMR of structure 7 with the protection of trimethyl silica.

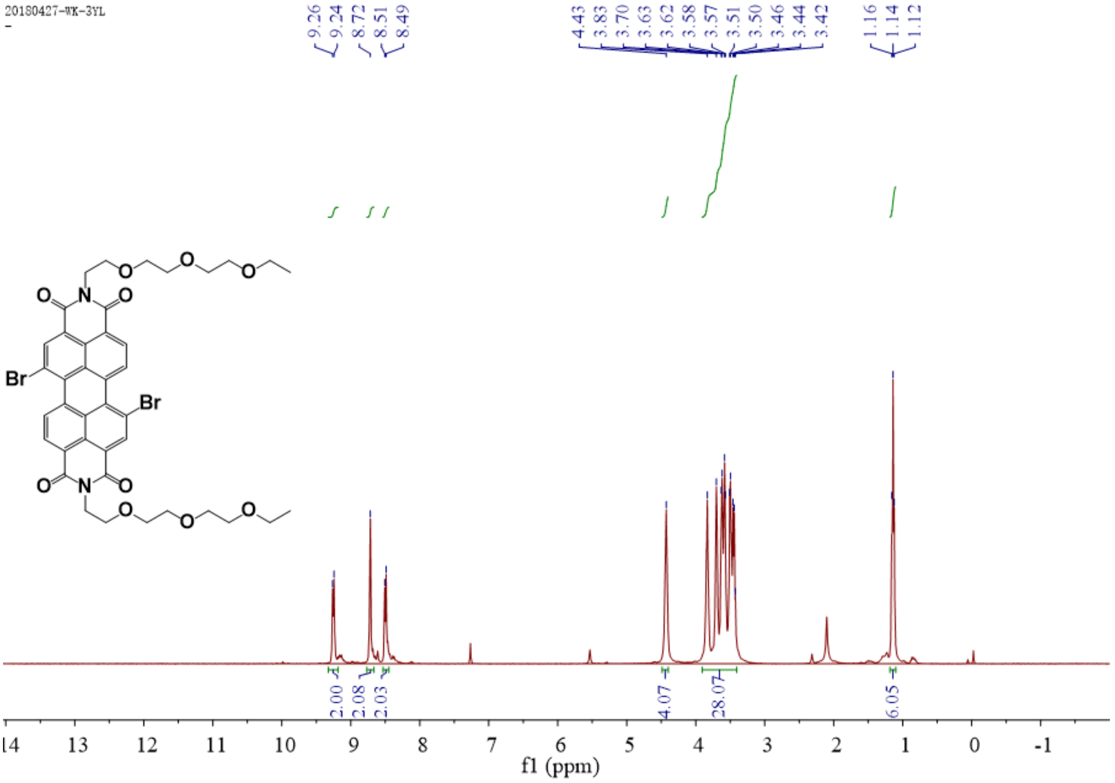

Figure S3. The ¹HNMR of structure 10.

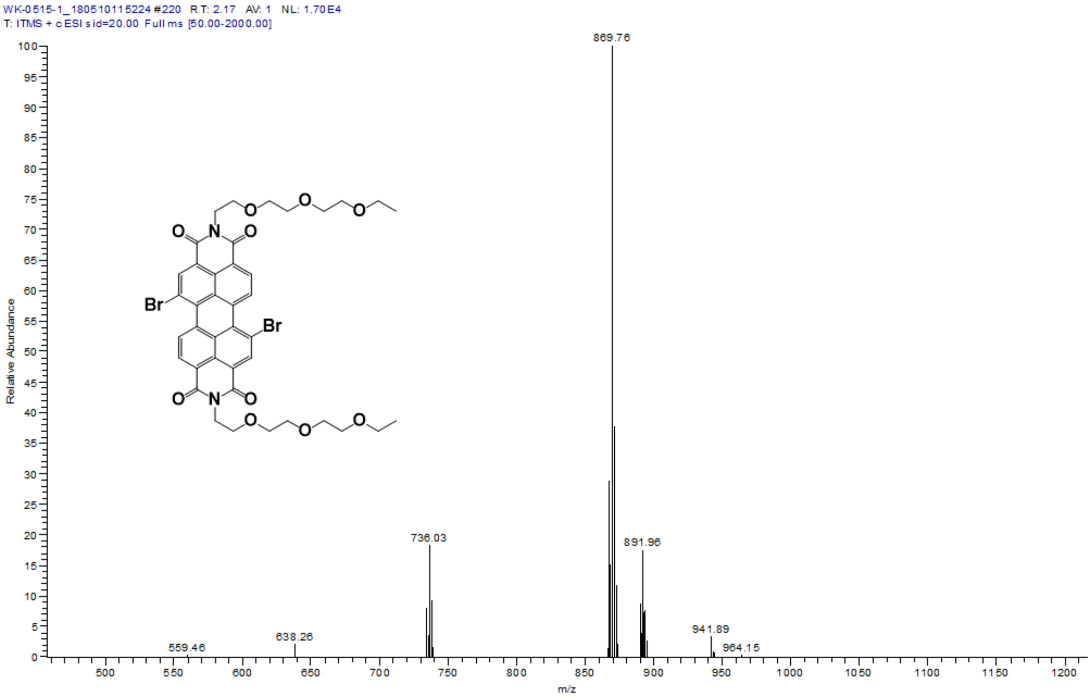

Figure S4. The ESI-MS of structure 9.

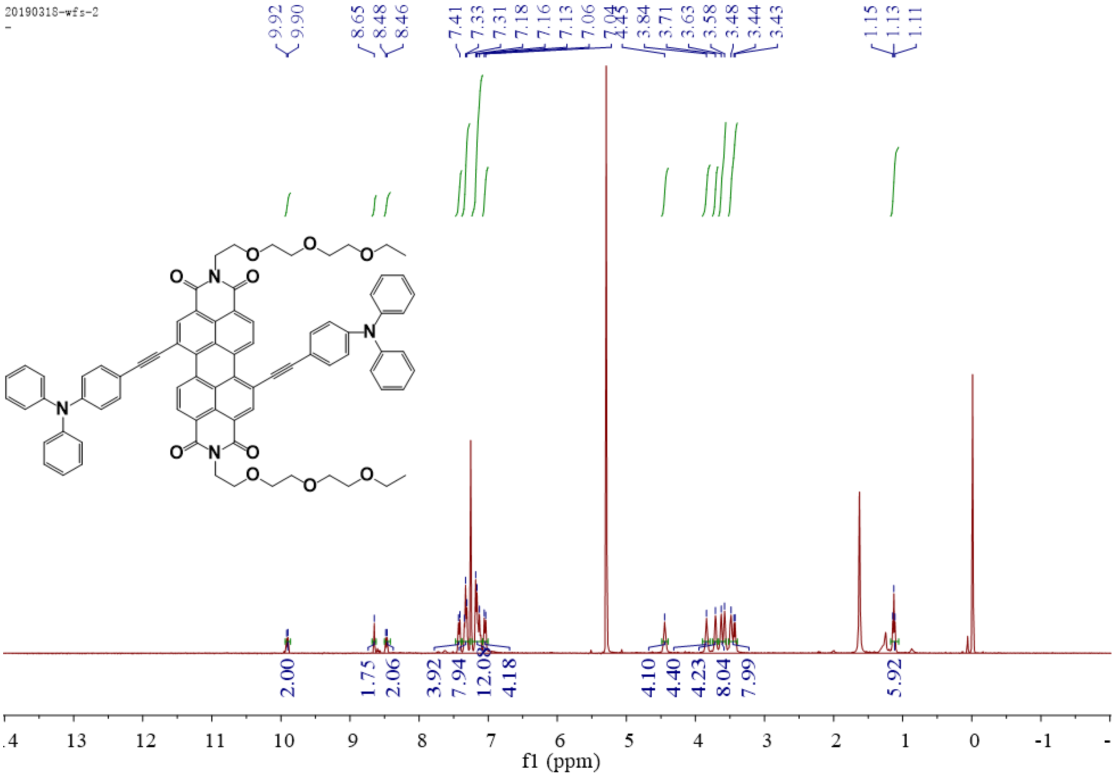

Figure S5. The ^{1}HNMR of PTA

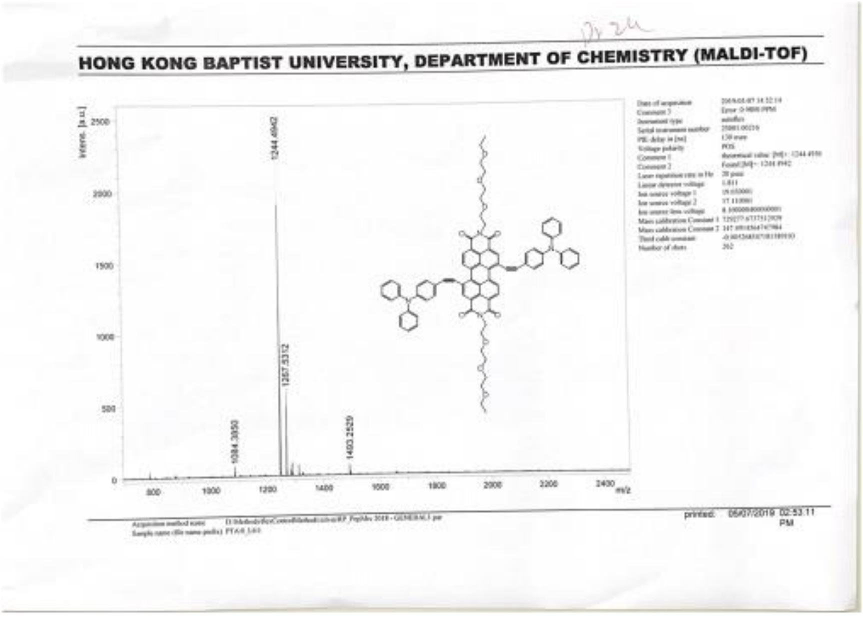

Figure S6. The MALDI-TOF of PTA.

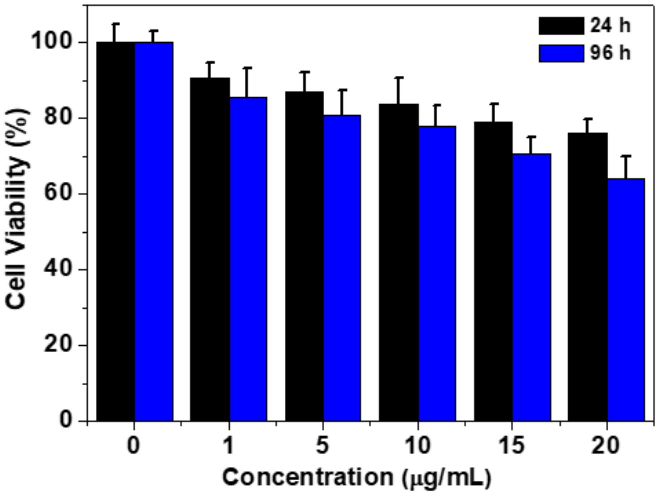

Figure S7. The cell viability of A549 cells incubated with different concentrations of TPA-NPs for a period of 24 and 96 h in the dark.

Reference

1. Alliband, A.; Meece, F.A.; Jayasinghe, C.; Burns, D.H. Synthesis and characterization of picket porphyrin receptors that bind phosphatidylglycerol, an anionic phospholipid found in bacterial membranes. *J. Org. Chem.* **2013,** *78* 356–362.
2. Rajavelu, K.; Rajakumar, P.; Sudip, M.; Kothandaraman, R. Synthesis, photophysical, electrochemical, and DSSC application of novel donor–acceptor triazole bridged dendrimers with a triphenylamine core and benzoheterazole as a surface unit. *New J. Chem.* **2016,** *40*, 10246–10258.
3. Mba, M.; D'Acunzo, M.; Salice, P.; Carofiglio, T.; Maggini, M.; Caramori, S.; Campana, A.; Aliprandi, A.; Argazzi, R.; Carli, S.; Bignozzi, C. A. Sensitization of Nanocrystalline TiO2 with Multibranched Organic Dyes and Co(III)/(II) Mediators: Strategies to Improve Charge Collection Efficiency. *J. Phys. Chem. C* **2013,** *117*, 19885–19896.
4. Huang, H.; Che, Y.; Zang, L. Direct synthesis of highly pure perylene tetracarboxylic monoimide. *Tetrahedron Lett.* **2010,** *51*, 6651–6653.
5. Yan, Q.; Zhao, D. Conjugated Dimeric and Trimeric Perylenediimide Oligomers. **2009,** *11*, 3426–3429.

6. Endres, A.H.; Schaffroth, M.; Paulus, F.; Reiss, H.; Wadepohl, H.; Rominger, F.; Kramer, R.; Bunz, U.H. Coronene-Containing N-Heteroarenes: 13 Rings in a Row. *J. Am. Chem. Soc.* **2016,** *138*, 1792–1795.
